# Multiligamentous Knee Injury in a Skeletally Immature Athlete

**DOI:** 10.5435/JAAOSGlobal-D-25-00266

**Published:** 2026-08-18

**Authors:** Jerome Murray, Shefali Bijwadia, Jacob Ristow, Bradley Nelson

**Affiliations:** From the Department of Orthopaedic Surgery, University of Minnesota, Minneapolis, MN (Dr. Murray, Dr. Ristow, and Dr. Nelson), and the Department of Orthopaedic Surgery, University of North Carolina, Minneapolis, MN (Dr. Bijwadia).

## Abstract

This case report details an adolescent boy aged 13 years 4 months who sustained a complete tear of the anterior cruciate ligament, posterior cruciate ligament, and medial collateral ligament with a partial lateral meniscus tear during a football injury. He underwent autograft anterior cruciate ligament reconstruction and allograft posterior cruciate ligament and medial collateral ligament reconstruction, followed by partial lateral meniscectomy. Postoperative rehabilitation included progressive range of motion and strength training, with return to sport achieved after surgery. At the 18-month follow-up, the patient reported no pain, had normal limb lengths, and demonstrated excellent functional outcomes, including return to competitive football. This case highlights successful multiligamentous knee reconstruction in a skeletally immature patient and underscores the importance of graft selection and surgical technique to minimize physeal injury and reduce the risk of leg-length discrepancy, allowing full return to competitive sports.

Multiligamentous knee injuries are uncommonly reported in the skeletally immature patient population. However, with increasing youth participation in organized sports, the incidence of ligamentous knee injuries in children is rising. Isolated pediatric anterior cruciate ligament (ACL) injuries have tripled since 2005, currently comprising up to 3% of all ACL injuries.^1^ Although extensive literature exists regarding ACL reconstruction (ACLR) techniques and outcomes in pediatric patients, reports addressing the surgical treatment of concomitant collateral and posterior cruciate ligament (PCL) injuries are rare.^2-6^ The goal of this case report was to contribute to existing literature regarding multiligamentous injury in skeletally immature patients.

We report the case of a patient aged 13 years 4 months with no notable medical or surgical history who sustained a complete tear of the ACL, PCL, and medial collateral ligament (MCL) as well as a partial lateral meniscus tear, following a football injury. He underwent ACLR with autograft, PCL and MCL reconstruction with allograft, and partial lateral meniscectomy. The patient returned to full participation in American football 18 months postoperatively, with no leg-length discrepancy (LLD) or other complications.

## Case Report

**Figure 1:**
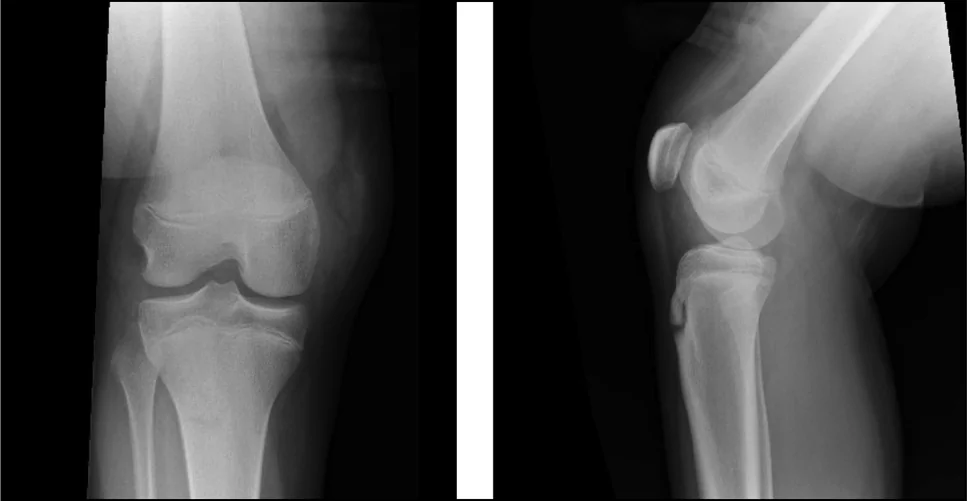
Anteroposterior and lateral radiographs of the right knee showing open distal femoral and proximal tibial physes.

This case was reviewed by the University of Minnesota Institutional Review Board, study number STUDY00019165. An adolescent boy aged 13 years 4 months injured his right knee while playing football, following direct impact to the lateral aspect of the knee. He reported immediate knee pain, swelling, and inability to bear weight. On initial physical examination, he exhibited grade 3+ effusion, passive range of motion from 0° to 60°, grade 2B Lachman, and a positive pivot shift. His PCL was difficult to examine. Posterior drawer was limited by notable guarding at 90° but was stable at 60° of flexion. No posterior sag was noted, and quadriceps strength was equal to the unaffected side. He had grade 3 laxity to valgus stress and was stable to varus stress at 0° and 30° of knee flexion. Radiographs displayed open physes of the distal femur and proximal tibia with no evidence of fracture or dislocation (Figure 1). Neurovascular examination was normal.

**Figure 2:**
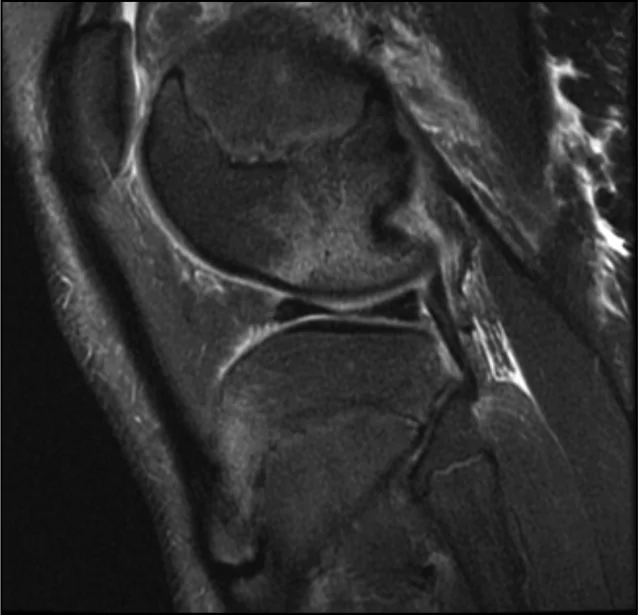
Sagittal MRI demonstrating PCL avulcion from the femoral attachement.

**Figure 3:**
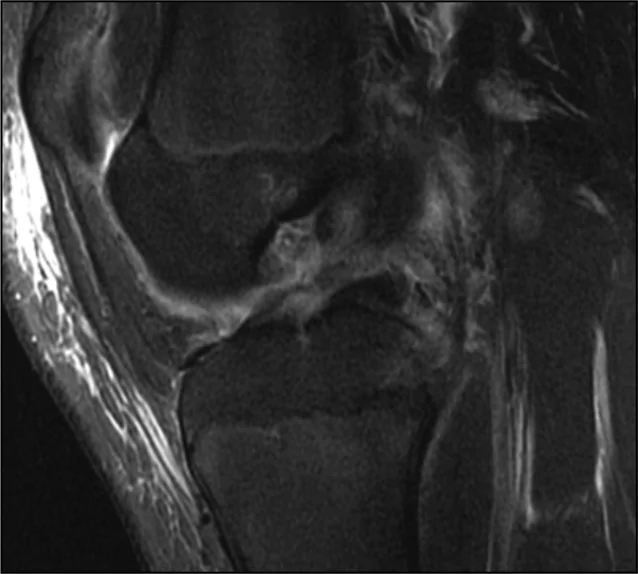
Sagittal MRI demonstrating midsubstance rupture of the ACL.

**Figure 4:**
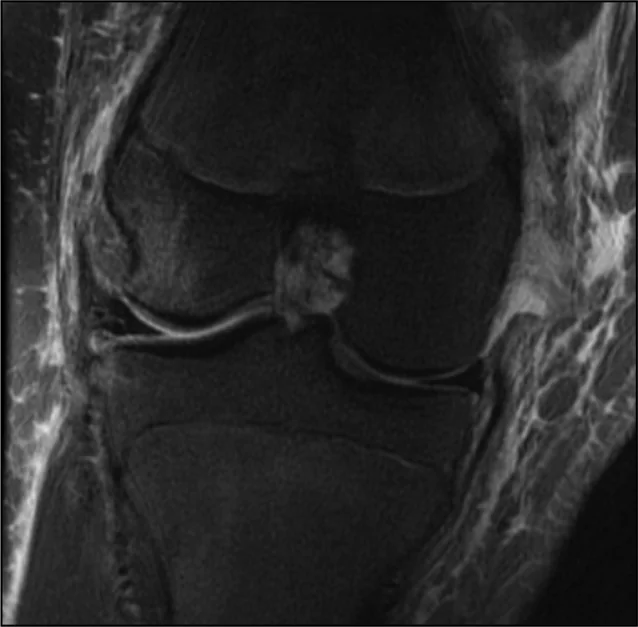
Coronal MRI demonstrating femoral avulsion of MCL and signal changes suggestive of a radial lateral meniscus tear.

Magnetic resonance imaging confirmed complete midsubstance rupture of the ACL, PCL from the femoral attachment, and an avulsion of the superficial MCL from its femoral insertion. In addition, T2 hyperintensity suggested a small radial tear in the midbody of the lateral meniscus (Figures 2–4). No injury of the extensor mechanism, hamstring tendons, or fibular collateral ligament or popliteus tendon was observed. There was concern for a potential injury to the meniscofemoral ligament of the medial and lateral meniscus

After an 11-week course of physical therapy, the patient displayed range of motion of 2° of hyperextension to 140° with a trace posterior drawer and 3 to 4 mm of increased laxity on valgus stress testing at 30°. Given these findings and subsequent discussion with the patient and his family, surgical intervention was recommended to address the ACL, MCL, and possibly the PCL and menisci.

## Surgical Technique

Standard anterolateral, anteromedial, and superomedial arthroscopy portals were established. A small radial tear involving the midbody of the lateral meniscus in the white-white zone was identified, consistent with the increased signal noted on preoperative magnetic resonance imaging, and was débrided back to a stable rim. Arthroscopic evaluation confirmed complete ACL and PCL ruptures. Owing to the condition of the ligaments, reconstruction of both was indicated. The cruciate ligaments were fully debrided, and a lateral wall notchplasty was performed. A posteromedial portal was created under direct visualization to facilitate removal of the tibial PCL stump and ensure clear access to the PCL's tibial insertion.

For the ACLR, a semitendinosus tendon autograft was harvested through a small anteromedial tibial incision. The graft, once quadrupled, yielded a final diameter of 9.5 mm. Both femoral and tibial fixation was achieved using an adjustable-loop cortical suspension device, incorporating a metallic retrograde cortical button for the femoral end. For PCL reconstruction, a presutured peroneus longus allograft was selected and prepared to a diameter of 9.5 mm. Fixation was similarly achieved using adjustable-loop cortical suspension constructs, mirroring the ACL configuration.

**Figure 5:**
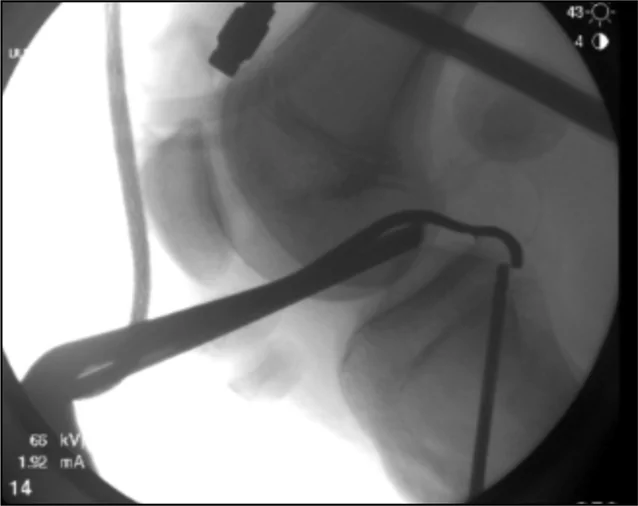
Intraoperative fluoroscopy showing closed-ended transphyseal tibial tunnel preparation using a retrograde drill for ACL and PCL reconstruction.

Under arthroscopic and fluoroscopic guidance, a 9.5-mm closed-ended transphyseal tibial socket was created for the PCL to a depth of 40 mm using a retrograde reaming technique. A similar tibial socket was prepared for the ACL (Figure 5).

**Figure 6:**
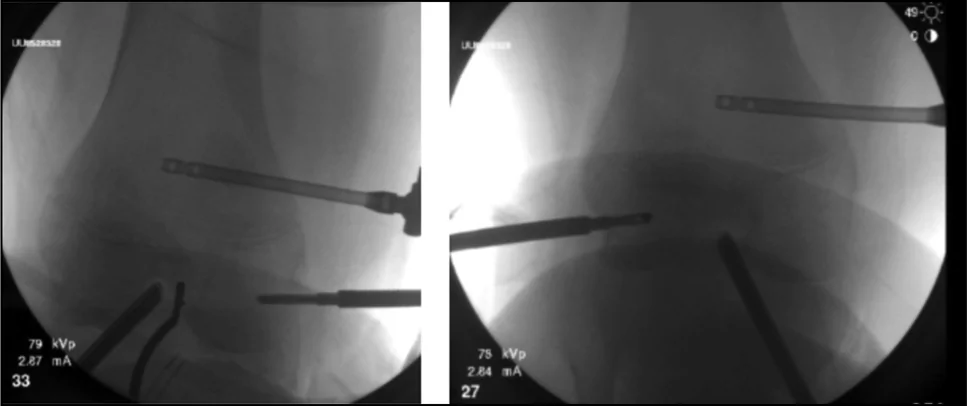
Drilling of the PCL (left) and ACL (right) all-epiphyseal femoral tunnels as confirmed with intraoperative fluoroscopy.

Epiphyseal femoral tunnels were created for both ligaments using a retrograde drilling technique. Fluoroscopy confirmed their all-epiphyseal tunnel placement to avoid distal femoral physeal disruption (Figure 6). Each tunnel was reamed to a depth of 25 mm, and shuttle sutures were passed accordingly.

The PCL graft was introduced through the anteromedial portal and positioned within the tibial tunnel under visualization from the posteromedial portal. The femoral end was then advanced into its tunnel, ensuring the cortical button was secured against the lateral femoral cortex. Appropriate graft positioning was verified fluoroscopically, and final tensioning was performed using the adjustable-loop mechanism. The ACL graft was subsequently passed and tensioned in a similar fashion. The graft was cycled through a full range of motion to confirm the absence of impingement. Both grafts were then secured using metallic cortical buttons.

For the MCL reconstruction, a separate longitudinal incision was made over the medial epicondyle. A guide pin was placed at the anatomical femoral origin of the MCL, with position confirmed fluoroscopically. A 7-mm reamer was used to create a short epiphyseal socket without breaching the distal femoral physis. A soft-tissue tunnel was then developed from the medial epicondyle to the prior hamstring harvest incision to facilitate graft passage.

A semitendinosus allograft was prepared with high-strength nonabsorbable sutures and passed in a retrograde fashion through the tunnel. The femoral end of the graft was secured using an adjustable-loop cortical suspension device with a retrograde cortical button, with appropriate positioning confirmed fluoroscopically. The tibial insertion of the MCL was identified, and a 4.5-mm screw with a spiked washer was anchored bicortically. The graft limbs were tensioned at 30° of knee flexion and secured under the washer.

**Figure 7:**
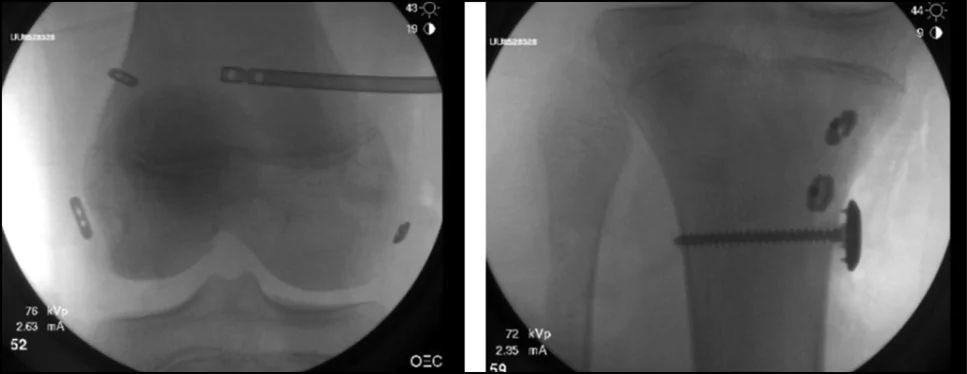
Final intraoperative fluoroscopic images showing cortical button fixation for the ACL and PCL (left) and washer fixation of the MCL graft on the tibia (right).

Final intraoperative examination demonstrated full range of motion with negative Lachman, a stable posterior drawer, and restoration of valgus stability at both 0° and 30°. Arthroscopic visualization confirmed appropriate graft tension, and fluoroscopy verified satisfactory implant positioning (Figure 7). The incisions were then closed in the standard fashion, and the patient was placed in a hinged knee brace for recovery.

Postoperatively, the patient initiated active and passive range-of-motion exercises from 0° to 90° in the brace beginning 2 weeks after surgery, consistent with the standard rehabilitation protocol for multiligamentous knee reconstruction. Toe-touch weight bearing was maintained until 6 weeks postoperatively, at which point the brace was discontinued, and weight bearing as tolerated was initiated. Low-intensity cardiovascular conditioning was introduced at 12 weeks, and the patient was transitioned to a functional knee brace. Jogging was permitted at 4 months postoperatively.

**Figure 8:**
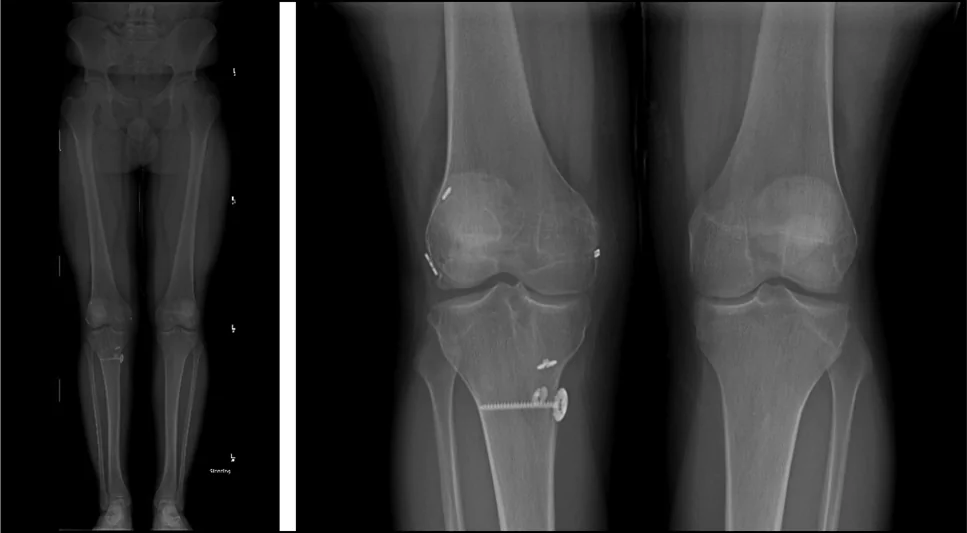
Standing leg length radiographs at 18 months postoperatively.

At the 1-year follow-up, the patient's Single Assessment Numeric Evaluation score was 95. He reported no pain or instability, with range of motion from 2° of hyperextension to 140° of flexion. Clinical examination and radiographs revealed no LLD or angular deformities in the coronal or sagittal plane at subsequent follow-up appointments (Figure 8). In addition, serial radiographs showed symmetric distal femoral condylar contours and a congruent tibial plateau without evidence of epiphyseal collapse, asymmetry, or contour irregularity, suggesting no disturbance of epiphyseal growth.

After 18 months of dedicated physical therapy, isokinetic strength testing revealed full recovery of quadriceps strength in the involved leg, matching or exceeding the strength of the uninvolved leg across concentric, eccentric, and isometric assessments. Physical examination revealed no effusion, negative Lachman, and negative pivot shift. Range of motion was from 2° of hyperextension to 140° of flexion. Hamstring strength on the involved side measured approximately 80% compared with the uninvolved leg, reflecting a mild but acceptable deficit relative to normative data. The patient was then cleared to return to competitive football without restrictions. At the time of this publication, the patient had no reported complications or limitations in activity, and no adverse events had occurred after their return to play.

## Discussion

Multiligamentous knee injuries in skeletally immature patients are uncommonly reported in the literature, with most studies focusing on isolated ligament injuries, particularly involving the ACL. Surgical techniques for ACLR in the pediatric population include all-epiphyseal reconstruction, transphyseal reconstruction, and hybrid or partial transphyseal reconstruction. When choosing a reconstruction technique, patient age and remaining growth potential must be carefully considered. Each technique carries the risk of LLD or angular deformities, although reported rates vary. One study noted a 16.7% incidence of growth disturbance after hybrid ACLR; however, all patients successfully returned to sport.^7^ Yoo et al^8^ reported 26% of patients with 5 mm or greater LLD after physeal-sparing ACLR. Hybrid and transphyseal reconstruction had similar rates of LLD, with overgrowth more frequently seen with hybrid reconstruction and growth arrest more commonly associated with transphyseal ACLR.^9-11^ A review by Wong et al^12^ of 45 studies involving 1392 surgically treated ACLs identified 58 cases of LLD, with 16 requiring corrective surgery.

Isolated PCL are also rare in pediatric patients and often present as osteochondral avulsion injuries.^2,13^ While nonsurgical treatment is the first line of management for most pediatric PCL injuries, surgical intervention is effective for those who fail conservative care or present with concomitant ligamentous injuries.^14^ Surgical techniques for PCL reconstruction in skeletally immature patients are similar to those for ACL injuries, including physeal-sparing, transphyseal, hybrid, and arthroscopic tibial inlay reconstruction.^15,16^ Numerous case studies report minimal LLD and no angular deformities after PCL reconstructions using Achilles or semitendinosus tendon allografts or autografts.^2,5,6,15,17^

In this case, we elected to perform transphyseal ACLR and PCL reconstruction with an all-epiphyseal MCL reconstruction. Careful surgical planning in this context involved deliberate pediatric-specific modifications aimed at minimizing physeal injury while maintaining stable multiligamentous reconstruction. Compared with adult reconstruction techniques, tunnel diameters were intentionally limited to the smallest size necessary to accommodate soft-tissue grafts, reducing the cross-sectional area of physeal violation. All reconstructions used soft-tissue grafts without bone plugs to avoid osseous fixation across active growth plates. Fixation strategies relied on cortical suspension rather than interference screw fixation, thereby eliminating implant placement within or across the physis. Fluoroscopy was routinely used to confirm epiphyseal tunnel placement and avoid unintended physeal breach, a step not routinely required in adult patients. Both ACL and PCL tunnels were created in a manner that minimized physeal damage, and the postoperative results demonstrated excellent functional outcomes without growth disturbances. Factors associated with growth arrest include fixation implant crossing the physis, tunnel diameter greater than 12 mm, and bone grafts traversing the physis.^14^ Techniques limiting physeal disruption to less than 7% of the cross-sectional area are protective against growth arrest.^3,18-23^

Although medial and lateral collateral ligament injuries are rare in children, existing literature suggests that multiligamentous injuries often require surgical intervention to achieve optimal outcomes.^24^ In select pediatric cases, primary repair of the MCL may be considered, particularly for acute femoral-sided avulsions with preserved tissue quality. However, in this patient, reconstruction was favored because of persistent valgus instability after prolonged nonsurgical management and concerns regarding tissue integrity. Reconstruction allowed for controlled epiphyseal graft placement while minimizing physeal risk. Similarly, although PCL avulsion repair represents a potential alternative treatment strategy, reconstruction was selected in this case because of poor tissue quality, limited bone stock, and the presence of chronic multiligamentous instability. Allograft ACLR was also considered; however, given higher reported failure rates in young, high-demand athletes, autograft reconstruction was chosen to optimize graft durability and long-term stability. To our knowledge, no reports exist of MCL reconstruction in skeletally immature patients.

## Conclusion

This case report describes a successful outcome in a skeletally immature patient with complex multiligamentous knee injuries. The patient returned to competitive sports without complications 18 months after ACL, PCL, and MCL reconstruction as well as partial lateral meniscectomy. This case contributes valuable insights into the management of multiligamentous knee injuries in young athletes and highlights the importance of surgical techniques that minimize the risk of physeal damage. The patient and their legal guardian were informed that the data concerning this case would be submitted for publication, and both provided their consent for the information to be used in this report.
